# Lived experience research as a resource for recovery: a mixed methods study

**DOI:** 10.1186/s12888-020-02861-0

**Published:** 2020-09-21

**Authors:** Anne Honey, Katherine M. Boydell, Francesca Coniglio, Trang Thuy Do, Leonie Dunn, Katherine Gill, Helen Glover, Monique Hines, Justin Newton Scanlan, Barbara Tooth

**Affiliations:** 1 School of Health Sciences, University of Sydney, Sydney, NSW Australia; 2 Black Dog Institute, Sydney, NSW Australia; 3 Mental Health Drug & Alcohol, Northern Sydney Local Health District, Sydney, NSW Australia; 4 St George and Sutherland Mental Health Services, South Eastern Sydney Local Health District, Sydney, NSW Australia; 5 Consumer-Led Research Network, Sydney, NSW Australia; 6 Enlightened Consultants, Brisbane, Qld Australia; 7 Upfront Leadership, Sydney, NSW Australia

**Keywords:** Lived experience research, Service user research, Knowledge translation, Mental health recovery, Hope

## Abstract

**Background:**

Lived experience research is conducted by people who have experience of mental health issues and is therefore better placed than more traditional research to illuminate participants’ experiences. Findings that focus on identifying enablers of recovery from a lived experience perspective have the potential to assist people in their recovery process. However, this lived experience research is often difficult to find, access and interpret. We co-produced user-friendly and engaging resources to disseminate findings from six lived experience research studies. This paper seeks to answer the research questions: a) Did exposure to lived experience research increase hopefulness for participants?; and b) How else did interacting with lived experience research resources influence participants’ lives?

**Methods:**

Thirty-eight participants were introduced to four resources of their choosing by peer workers over a four-week period. The helpfulness of resources was evaluated using mixed methods, including a quasi-experimental analysis of change in hope, an anonymous survey and in-depth interviews.

**Results:**

Findings indicated that the resources promoted hope, but that increases in hopefulness may not be seen immediately. Other impacts include that the resources: encouraged helpful activities; provided a positive experience; increased valued knowledge; encouraged people to reflect on their journey and think constructively about mental health issues; helped people to feel less alone; and assisted people to explain their situation to others.

**Conclusions:**

The research suggests the potential usefulness of lived experience research resources, presented in user-friendly formats, in the lives of people who experience mental health issues and implies a need to nurture this type of research.

## Background

Lived experience research in mental health is research that illuminates the perspectives and experiences of people who live with mental health issues and is conducted either by researchers with their own lived experience or in collaborative research teams that include people with lived experience [1, 2]. This paper investigates the usefulness of lived experience research in the lives of people living with mental health issues.

The importance of lived experience research in mental health is increasingly recognised and usually conceptualised in terms of three major benefits. First, consumer rights activists, using the slogan of “nothing about us without us” have argued that inclusion in research is a human right and a social justice issue [3]. Second, it can produce better quality research by enhancing methodological sensitivity, data accuracy, validity of results, and overall relevance to service users e.g., [4–6]. Third, people with lived experience have reported deriving benefits from doing research such as satisfaction, skill development, empowerment, and hope [4, 6]. Lived experience researchers are increasingly adopting leading roles in conceptualising and conducting research in mental health.

Findings from lived experience research have the potential to be helpful to people in their recovery journeys. Numerous studies have reported the benefits of learning from the wisdom, strategies, challenges and successes of others e.g., [7]. Hope, a critical component of recovery [8], is also a major benefit of being exposed to the stories and experiences of others in similar situations. A recent study examined the types of experiences that people living with mental health issues described as igniting and maintaining hope [9]. Two sources of hope were particularly relevant to lived experience research. First, hearing positive stories of others’ experiences was important. As one participant stated: “the consumers’ voice was hope and healing”. Second, hope was promoted by learning gained from others with lived experience, such as “the key tips and strategies that other peers discussed.”

Observing peers who are living well and reading or listening to individual narratives of recovery are important ways in which people learn from each other and derive hope. However, lived experience research has the potential to bring together the stories of a variety of different people to provide a range of ideas and a bigger picture on particular issues, thus contributing to an individual’s store of resources for recovery.

While the researchers were unable to locate research about the direct use of lived experience research by people living with mental health issues, our collective experience has indicated that many who are not themselves involved in user-led or collaborative research, do not even know that it exists, let alone how to access the findings. Little is known, therefore, about how useful people might find lived experience research in their daily lives.

Our research team, consisting of researchers with and without lived experience of mental health issues, set out to address this issue. As research is rarely presented for a lay readership, we developed a range of user-friendly formats to disseminate lived experience research findings to people living with mental health issues.

This paper seeks to answer the following research questions:

a) Did exposure to lived experience research increase hopefulness for participants?

b) How else did interacting with lived experience research resources influence participants’ lives?

## Methods

### Study design

We collaborated with peer workers and final year design students to develop a suite of six lived experience research resources. These were introduced to consumers by peer workers, and the intervention was evaluated using a mixed methods approach. A mixed methods approach enabled the research questions to be addressed from different perspectives, providing a fuller picture than could be gained using a single method [10, 11]. A quasi-experimental evaluation of hope sought to provide relatively objective evidence of the impact of the intervention; an anonymous survey provided comparable participant ratings of the intervention’s impact in expected areas; and qualitative interviews enabled inductive identification of experiences of most importance to participants. Ethical approval was obtained from the LHD’s Human Research Ethics Committee. Reporting adheres to guidelines for Good Reporting of A Mixed Methods Study (GRAMMS) in health service research [12, 13].

### Resources

We reviewed the literature to identify lived experience research papers in which the findings were directly relevant to the daily lives of people living with mental health issues. We consulted with peer workers and others with lived experience to identify topics most likely to be of interest to users. Through these processes, we identified six research studies to develop into user-friendly resources. Translating these began with a conference workshop [14] and a full day design lab focused on design thinking [15]. These were attended by service users, peer workers, researchers, clinicians and final year design students from the University of Technology Sydney. After the design lab, the ideas and prototypes were taken up by the design students for further development. They designed and produced the resources with regular input on content and format from the research team and peer workers. The resources are summarised in Table 1. Detailed descriptions and photographs are provided in the supplementary materials.

**Fig. 1:**
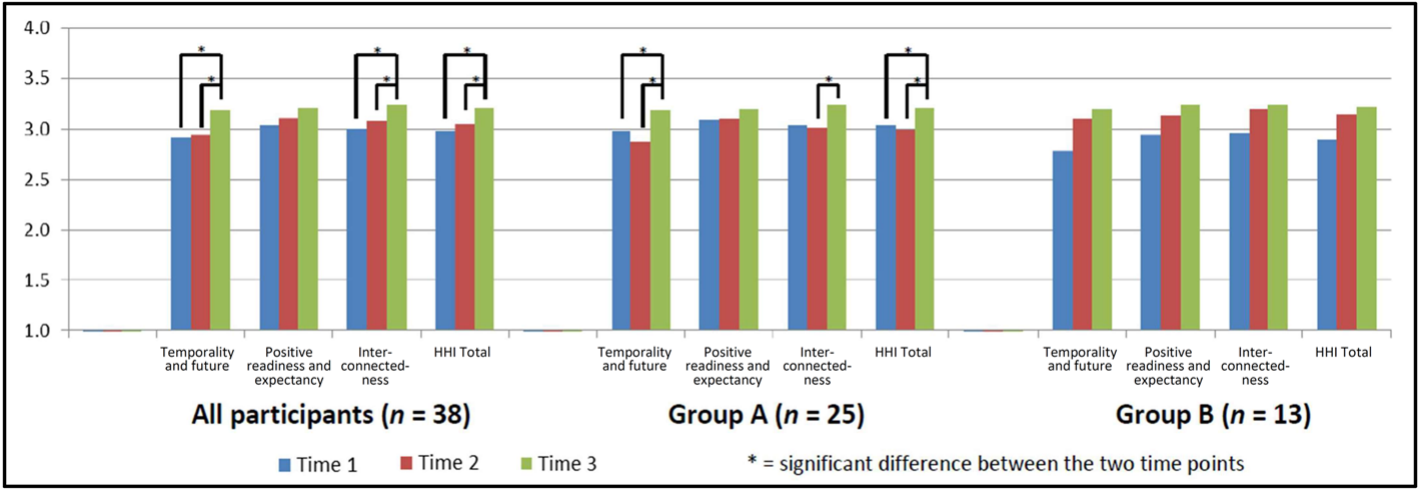
Change in Herth Hope Index over time: all participants and by group allocation

**Table 1:** Resources

Topic	Reference	Format
concepts of recovery	Factors consumers identify as important to recovery from schizophrenia [16]	Podcast of interview with authors
what helps recovery	Mental health recovery: What helps and what hinders? [17]	Portraits with handwritten quotes and explanation of themes
personal medicine	The importance of personal medicine: A qualitative study of resilience in people with psychiatric disabilities [18]	Workbook in Webster pack format
hope	Igniting and Maintaining Hope: The Voices of People Living with Mental Illness [9]	Personalisable “Hope box” containing paper cranes and hopeful quotes.
physical health care	Mental health consumer experiences and strategies when seeking physical health care: A focus group study [19]	Card deck with graphically designed matching cards illuminating 11 themes.
meaningful activity	Coping with mental health issues: Subjective experiences of self-help and helpful contextual factors at the start of mental health treatment [20]	Magazine about different types of meaningful activities and how people used them.

### Intervention

During peer worker training, each of the finalised resources was examined by peer workers and the research team, who together reached consensus on how each resource would be introduced to consumers. This was flexible however, enabling peer workers to adapt their explanations and activities to be most appropriate to the needs of individual participants. The agreed upon protocols were developed into a peer worker manual.

In recognition that different content is relevant to different people, participants were asked to select four of the six resources. Peer workers introduced participants to one resource per week for 4 weeks. For most resources, the peer workers showed each participant the resource, went through some of it in detail, explained how it was designed to be used, then gave it to the participant to keep and use in whatever way they preferred.

### Sampling and recruitment

#### Site

The project was carried out in one Local Health District (LHD) in Sydney, Australia. The LHD employs 18 peer workers over three inpatient and four community sites. The project employed five of these peer workers to recruit and provide the intervention to clients of these services. Contacts between peer workers and participants took place wherever peer workers normally met with their clients, for example on an inpatient unit, at a community mental health service, or in a community venue such as a coffee shop.

#### Criteria

Eligible participants were: clients of the LHD; able to speak and read English; and able to provide informed consent. Clients were excluded if they were considered by their peer worker or primary clinician to be unable to fully understand the procedures, risks and benefits of participation due to acute illness. We planned to recruit 30–40 participants as previous research indicated that this sample size was sufficient to show change [21].

#### Recruitment

Peer workers explained the study to all eligible clients that they saw in the course of their work. If a client was interested, the peer worker gave them written project materials (flyer, participant information sheet and consent form), offered to read through the forms with them, and answered any questions. Clients were given several days to read and think about the project and were invited to call the Chief Investigator to discuss the project further if they wished. In several days, the peer worker recontacted the client and, if they wished to participate, obtained written informed consent. Peer workers emphasised that the research was voluntary, participants could withdraw at any time, and participation or refusal would have no impact on their other interactions with peer workers or health service. Consent was considered not as a one-off event, but an ongoing negotiation between peer workers and participants [22], where the primary concern was participants’ well-being. Therefore, at each research-related interaction, peer workers obtained verbal confirmation that the client was happy to continue taking part. Participants were given a $50 gift voucher after study completion to thank them for their time.

#### Allocation to groups

After providing informed consent, participants were allocated to group A or group B to determine when they would receive the intervention. In most cases this was done using a coin toss, however, the staggered timing of recruitment and other peer worker commitments made it necessary for 13 participants to be allocated based on logistical issues. This also meant that the groups were uneven, with 25 participants allocated to group A and 13 participants allocated to group B.

### Data collection

Hopefulness was measured using the Herth Hope Index (HHI). The HHI is a 12-item scale that was developed for clinical populations, takes just a few minutes to do, has good psychometric properties [23] and has been used with a variety of different clinical groups in at least seven languages e.g., [24]. It includes three factors of: *temporality and future*; *positive readiness and expectancy*; and *interconnectedness*. Participants completed the HHI at three timepoints. Group A received the intervention between T1 and T2; group B received the intervention between T2 and T3.

Participants were asked to complete an anonymous online evaluation survey once only, after they had received their four resources (at T2 for group A and T3 for group B). This consisted of a series of fixed-choice questions about each resource including its impact on various aspects of participants’ lives and their overall experience of the project.

Semi-structured interviews [25] were conducted after participants had received the resources and completed T2 (group A) or T3 (group B). They were conducted by Author 8, who had not been involved in the intervention. An interview guide was used containing open ended questions. The interview guide was used flexibly, allowing for conversational flow and follow-up questions to gather detail about issues that were of importance to participants [25]. Participants were asked for feedback on the individual resources and about the impact of the resources on them. Questions included: ‘Do you think you got any benefits out of being a participant in this study?’ ‘Was there anything that you didn’t like about being in the study?’ and ‘Did anything change for you as a result of engaging with the resources?’

Interviews were conducted in person in a private room in the health service or, where the participant preferred, over the phone. Interviews lasted between 7 and 30 min, averaging 17 min. Interviews were audio recorded and transcribed verbatim for detailed analysis. Participants were provided with both a copy of their transcript and a summary of findings and invited to comment, however, no participants provided additional feedback.

### Data analysis

#### Herth Hope index

Total scores were calculated for each factor (*temporality and future*; *positive readiness and expectancy*; and *interconnectedness*) and the overall total score. To examine change over time, paired *t*-tests were completed between Time 1 and Time 2; Time 2 and Time 3 and Time 1 and Time 3 for all participants as well as for Group A and Group B participants separately. Statistical analysis was conducted using SPSS.

#### Anonymous survey

Frequencies were calculated and presented in visual format to understand the range of responses.

#### Qualitative interviews

Data from participant interviews were analysed using interpretative content analysis (ICA). This hybrid method combines qualitative and quantitative techniques [26, 27], enabling inductive identification of themes as well and reporting of the frequency of those themes [26, 28].

The first step in ICA is inductive coding. Constant comparative analysis (CCA) was employed, as it is a systematic, rigorous, and well-established coding technique which minimises the risk of omission of data (Charmaz, 2014). Segments of data, such as phrases or sentences were examined and allocated one or more code names to reflect the underlying concepts they represented. Each new segment of data was compared to others to identify underlying similarities. For example, the data segments ‘just because you are unwell at times doesn’t mean staying unwell all the time’ and ‘Hope changed for me, it gave me a different angle of hope’ were found to represent the same concept: gaining hope. New data were also compared to existing codes and either added to these, or new codes were developed. Codes were compared to each other and refined by merging similar codes or grouping codes into higher level categories. NVivo computer software [29] was used to manage the data. Authors 1 and 4 independently coded the first three interviews, then met to discuss coding decisions and reach consensus. Thereafter, the authors met regularly to discuss and review coding decisions. These discussions were aimed at enhancing interpretive rigour, ensuring participants’ viewpoints were faithfully represented. When all interviews had been coded, and the coding list finalised, the transcripts were re-examined to ensure comprehensive coding [26]. NVivo was then used to identify the number of participants who discussed each theme.

#### Integration

When data from each component of the study had been analysed, the findings were compared to each other. Authors responsible for analysing different sections (primarily authors 1, 4 and 9) presented findings to the other authors and, through close discussion, questioning, and returning repeatedly to the data, derived an integrated interpretation of the results.

## Results

### Participants

Sixty-four people were invited to be part of the study and 43 agreed to participate. Five participants (2 from group A and 3 from group B) withdrew from the study after the first assessment and did not receive any of the resources. No participants withdrew between receiving the first resource and the post intervention assessment. Participants were not required to provide explanation for not participating or withdrawing but reasons mentioned included: “limited time/too busy”; “not interested”; “couldn’t be bothered”; “school commitments”; “mental health is okay”; and “anxious”. Thirty-four completed all three assessments, while four participants completed only the pre and post intervention assessments. Thirty participants completed the anonymous survey and 33 participated in the qualitative interviews. Table 2 presents the characteristics of people who participated in the study (*n* = 38).

**Table 2:** Characteristics of participants

Variable	Variable values	n (%)
Gender	Male	13 (34%)
	Female	24 (63%)
	No response	1 (3%)
Country of birth	Australia	31 (82%)
	Other (1 each from Bangladesh, Iraq, Malaysia,	7 (18%)
	New Zealand, Papua New Guinea, Peru and	
	Taiwan)	
Primary language spoken at home	English	30 (79%)
Marital status	Married/co-habiting	2 (5%)
	Unmarried	30 (79%)
	Separated/divorced	6 (16%)
Indigenous status	Aboriginal and/or Torres Strait Islander	2 (5%)
Recruitment source	Acute inpatient unit	1 (3%)
	Rehabilitation inpatient unit	1 (3%)
	Community service	36 (94%)
Education	Did not complete high school	5 (13%)
	Completed high school	7 (18%)
	Trade/technical/vocational training	6 (16%)
	Some college or university	4 (11%)
	Bachelor’s degree	8 (21%)
	Postgraduate certificate or diploma	8 (21%)
Employment status	Employed (paid)	8 (21%)
	Unemployed	30 (79%)
Currently studying	Recovery college courses	8 (21%)
	Bachelor’s degree or diploma	3 (8%)
	Certificate 2, 3 or 4	4 (11%)
	Other	1 (3%)
Duration of mental health issues	< 1 year	3 (8%)
	1–3 years	3 (8%)
	4–6 years	2 (5%)
	7–10 years	5 (13%)
	> 10 years	25 (66%)
Diagnoses^a^	Schizophrenia and other psychotic disorders	22 (58%)
	Depressive disorders	9 (24%)
	Personality disorders	1 (3%)
	Trauma and stressor related disorders	3 (8%)
	Bipolar and related disorders	7 (18%)
	Anxiety disorders	5 (13%)
	Obsessive compulsive and related disorders	1 (3%)
	Eating disorders	1 (3%)
	Did not answer	5 (13%)

^a^ 13 participants reported 2 or 3 diagnoses

While our intention was to recruit participants from inpatient and community settings, 36 of the 38 participants were living in the community. This was due to logistical and staff issues rather than potential inpatient participants declining.

The findings are presented below for each of the two research questions. During analysis, the impact of the research context emerged as a factor to be considered in the interpretation of the other findings. Therefore, findings around this issue are also presented.

### Does exposure to lived experience research increase hopefulness?

Data about the impact on hopefulness of engaging with the resources comes from all three data sources: the HHI, anonymous survey, and qualitative interviews.

#### Herth Hope index

Participant responses to the HHI are summarised in Fig. 1. There were no significant differences between Time 1 and Time 2 for Group A. However, significant improvements were seen in *temporality and future* (t = 3.4; *p* = 0.003), *interconnectedness* (t = 2.7; *p* = 0.013) and HHI Total Scores (t = 3.1; *p* = 0.006) from Time 2 to Time 3 and in *temporality and future* (t = 2.3; *p* = 0.030) and HHI total (t = 2.6; *p* = 0.019) from Time 1 to Time 3. There were no significant differences between time points for Group B. For the combined data set, significant improvements were seen in *temporality and future* (t = 3.1; *p* = 0.004), *interconnectedness* (t = 2.5; *p* = 0.018) and HHI Total Scores (t = 3.1; *p =* 0.004) from Time 2 to Time 3 and in *temporality and future* (t = 2.8; *p* = 0.008), *interconnectedness* (t = 2.2; *p* = 0.035) and HHI total (t = 2.4; *p* = 0.023) from Time 1 to Time 3 (Fig. 1).

#### Anonymous survey

**Fig. 2:**
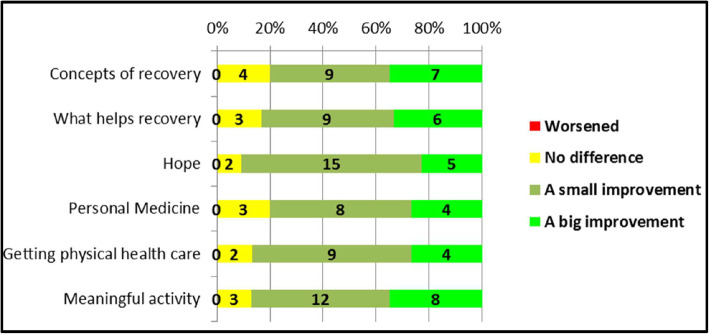
Has accessing the resource made a difference in your life in terms of your beliefs about your future or recovery?

In the anonymous survey, between 80 and 91% of participants who chose each resource reported that it had caused some improvement in their beliefs about their future or recovery, indicating an increase in hope. Responses for each resource are shown in Fig. 2.

#### Qualitative interviews

In the qualitative interviews, more than half of the participants (17/33) described how interacting with the resources made them feel more hopeful, positive and empowered. For example, P13 stated, regarding the meaningful activities magazine, that “I just had a little bit of a light bulb moment saying, ‘Well, these things help these people feel better and all these ideas’, so it gave me a bit of understanding and hope for my future.” Similarly, P12, commenting generally about the resources, said that “Seeing other people’s experiences, and that’s really helped to know ‘I can do that too’.” It should be noted that participants were not asked about hope specifically; the theme of hope emerged in response to general questions about the impact of the resources.

### How else did interacting with lived experience research resources influence participants’ lives?

**Fig. 3:**
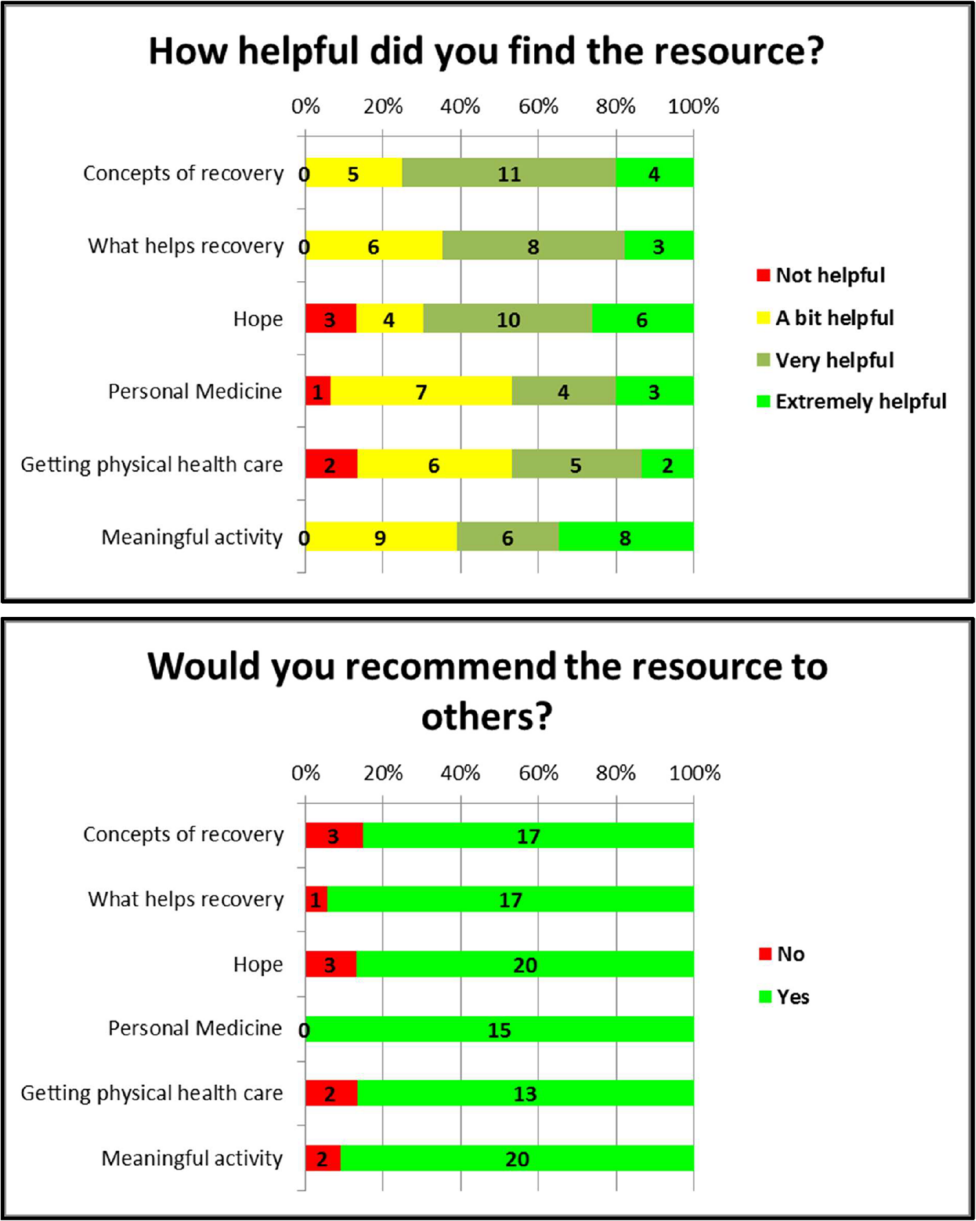
Participant perceptions of resources

It can be seen from Fig. 3 that an overwhelming majority of participants in the anonymous survey found each of the resources helpful, with between 46 and 75% of people finding each resource ‘very helpful’ or ‘extremely helpful’. Further, between 85 and 100% of people, depending on the resource, said that they would recommend it to other people.

**Fig. 4:**
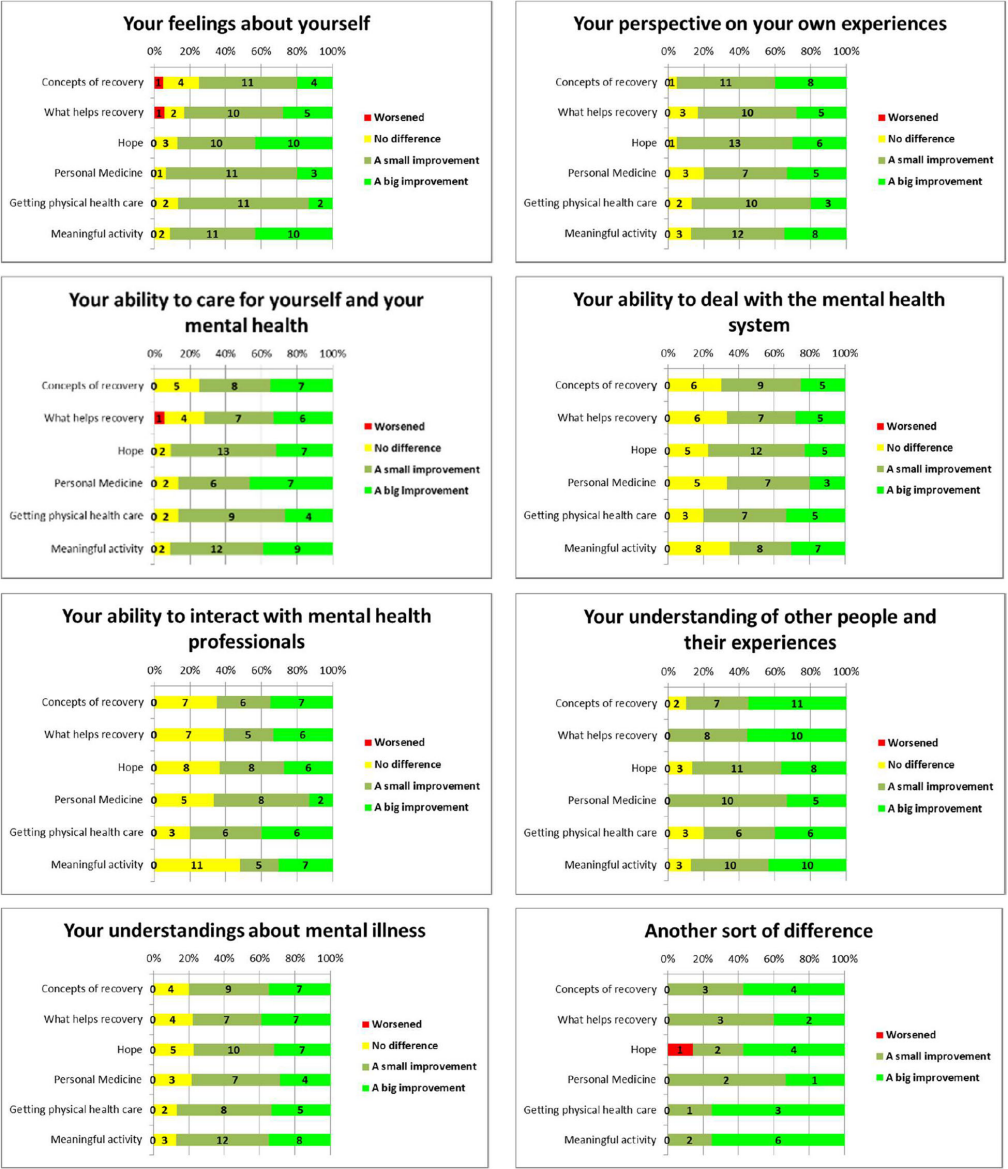
Has accessing the resource made a difference in your life in terms of

Participants also indicated that they perceived a positive impact of the resources on the specific aspects of their lives that were measured. For each resource, 60 to 80% of participants reported it had made a small improvement or a big improvement in their lives. Results are summarised in Fig. 4.

The in-depth interviews allowed participants to state their perspectives on the impact of the resources that mattered to them. Overall, 30/33 participants stated, when asked specifically, that they had benefited from being part of the study. Of the remaining three, two answers were unclear and one was not sure if they had benefited. This participant, P31, also reported that nothing had changed for them as a result of the study. With the exception of P31, all other participants described some positive impact from interacting with the resources in subsequent discussion.

The positive impacts people described fell into a number of broad categories, described and exemplified in Table 3. Counts are the total number of people who mentioned experiencing this impact. As noted above, these impacts emerged as responses to open questions, so a participant not mentioning an impact does not guarantee that they did not experience it.

**Table 3:** Self-perceived positive impact of resources

Impact	Example quote	***n*** (%)
Motivated helpful activities	Participants often talked about the resources prompting or motivating them to do things that were beneficial for them. This was through reminding them of helpful strategies, suggesting new strategies, or showing what had helped other people. *I’ve been a bit unwell, so I’m scaling back my hours and it’s the reason for that is basically I don’t have the time to do the self- care things I need to do. I really connected with that book because as I was reading it and I thought “These are the things I need to do”. [Meaningful activities – P04]* *I knew these sorts of things anyway, but it was just a matter of when I was going to activate them in my head, to sort of act on them... [the resources] did, they prompt you. [General comment – P14]*	**20 (61%)**
Positive experience in the moment	A number of participants talked about how interacting with the resources was an enjoyable or interesting experience at the time. *The little booklet’s very relaxing. [Meaningful activities – P24]* *The first podcast was interesting to listen to. [Concepts of recovery – P10]*	**19 (58%)**
Gaining knowledge	Participants talked about gaining new knowledge from the resources, such as information about mental health issues and strategies to try. *Just opening my eyes to a lot of things I didn’t know about [General comment – P13]* *I got a lot of ideas and suggestions that really made sense to me, such as how to stay well, when to recognize you’re not feeling well. [General comment – P13]*	**18 (55%)**
Reflecting on my journey	Commonly, people talked about how engaging with the resources made them reflect on their own experiences, strengths and journey. *It does make you think about what you’ve gone through. [General comment – P23]* *I understand myself a little bit better with what’s going on. [General comment – P24]*	**17 (52%)**
Thinking constructively about mental health issues	Participants discussed how the resources reinforced or promoted positive or useful perspectives and ways of thinking about and conceptualising mental health issues. *There was a few things that stood out and actually made me think that mental illness is not something that you should be really afraid of. [General comment – P13]* *It makes my belief in recovery much stronger. [General comment – P18]*	**15 (45%)**
Feeling less alone	Ten people noted that interacting with the resources made them feel that there were people who shared their experience and who understood them, which made some feel less alone and more supported. *I would say that I did feel a sense of support. I haven’t felt that really, like I’ve felt it with friends, but to feel more understood, more just that people understood, and people that do care, that people’s mental health experiences and are living well. [General comment – P15]* *I just kind of feel like they cared for me. So it was quite good getting the resources and then realizing that you’re not alone as well. There’s other people going through things as well. [General comment – P32]*	**10 (30%)**
Explaining to others	Some people talked about how they were able to use the resources to start conversations with others. For some, this was about using the resources and information to help others. For other people, the resources helped them to explain their condition or experiences to other people so that they understood better. *It’s learning different key words that I could use to say the same thing, so that it is more understandable for more people. [General comment – P06]* *Well, I showed them to my mother, who’s living with me at the moment, and I think it gave her a little bit of insight. [General comment – P08]*	**7 (21%)**

#### Negative impacts

While most of the impacts participants described were, as seen above, very positive, a few participants reported negative impacts. In the anonymous survey, three people reported that accessing a specific resource had a negative impact in one or two of the specified areas, as seen in Fig. 4. To contextualise these responses, they were considered alongside each participant’s responses to other questions and are reported in Table 4.

**Table 4:** Participants reporting negative impacts in the anonymous survey

Participant	A	B	C
Resource	Concepts of recovery podcast	What helps portraits	Hope box
Aspects worsened	Feelings about self	Feelings about self; ability to care for self and mental health	Another aspect (did not specify)
Aspects improved	Beliefs about future or recovery; ability to deal with the mental health system; understanding of other people and their experiences.	Perspective on own experiences; understanding of other people and their experiences.	Feelings about self; beliefs about future or recovery; ability to care for self and mental health; understandings about mental illness; understanding of other people and their experiences.
Overall rating of the resource	Very helpful	Very helpful	Very helpful
Would they recommend the resource to others	No	Yes	Yes
Overall experience of participating in the study	Very positive	Quite positive	Very positive
Other comments	n/a	‘keep up the research’	‘I liked being part of this program!’

The qualitative interviews also revealed some negative impacts of the resources and provided more detailed information. Three participants reported experiencing some distress from interacting with the resources. It is not possible to tell whether these are the same participants who reported the negative impacts in the anonymous survey. Two participants, while reporting a positive overall experience with the project, said that they had found the content of specific resources distressing because of their past experiences and life circumstances.

P26: Just some of the recommendations [from the hope box] felt like a stab in the gut. Something that I couldn't do in my own life … the one about spending time with friends because I felt that I'd lost friends during my hospital stay*.*

P19: Personal medicine was, I didn't want to use at all. I just didn't anticipate it. I just, I actually had an upset because I'm an astrologer. I have my own personal way of looking at life … I don't want to have more psychology stuff*.*

The third participant described feeling upset from hearing about other people’s experiences but did find them ultimately hopeful.

P13: Some of what the participants were experiencing, I experienced those symptoms and I thought it is upsetting. But with what they've set their hope in things to do, it also made me think, well, then I can still feel hopeful about the future*.*

### Impact of the research context

Participants in the qualitative interviews reported that they enjoyed being part of the research project. Findings from the anonymous survey supported this; in response to the question “Overall, how would you describe your experience of participating in the study?”, 18 participants (60%) reported a very positive experience, 10 (33%) gave a ‘quite positive’ response, and two (7%) were neutral. No participants reported a negative experience.

These positive experiences may not, however, have been about the resources alone. Ten people specifically mentioned that they had found being part of the research process a valuable and affirming experience. They appreciated being asked for their opinions about the resources and valued being able to contribute to a piece of research that they saw as worthwhile. Some reported being pleased to know that people with lived experience were doing research and found this hope inspiring.

P25: I really valued being able to, like, participate and do something worthwhile*.*

P15: I felt stronger because of it, like there's people that care and people that are making an effort to try and help and improve the lives of others*.*

P26: I think it's helped a lot with my recovery. Engaging with the materials and trying to make them the best that they can be*.*

The responses of fourteen additional participants to a question about what had motivated them to be in the study, also suggested positive feelings about the research process. Six of these reported that they had agreed to participate in the project because of a desire to make a positive contribution to their community and to the mental health system, saying things like “I felt that maybe I can make a difference for other people like me” (P28). A further six were attracted to it as a piece of research. P29, for example “was interested in the type of research”, while for P27 it was “because I believe in research”. Two more participants wanted their voice to be heard, saying, for example: “I thought it would be good to sort of have my own opinion put out there” (P20).

Four participants spontaneously expressed the hope that the project would continue into the future.

P14: I just hope something, you guys are able to elaborate on, give more of it, the research, to people. I think it's really good, because it could save someone's life. So, I just think, just keep going with it*.*

## Discussion

This study is the first to examine the potential impacts of accessing lived experience research for people living with mental health issues. Overall, the findings suggest that lived experience research, presented in accessible formats, can result in positive experiences and outcomes.

Initially, the results obtained from the HHI appeared counter-intuitive. The original hypothesis was that participants would demonstrate improved hope between times 1 and 2, for group A and between times 2 and 3 for group B (i.e., that hope scores would increase immediately after engaging with the resource). This was not the case. Yet results from both the anonymous survey and the qualitative interviews indicated that many participants did find engaging with the resources to be hope inspiring. The significantly increased HHI scores between post-intervention and follow-up for group A could suggest that more time is required before the impact of the resources is seen in relation to hopefulness, possibly to integrate learnings from the resources into everyday life. Perhaps if group B had completed the HHI a month following engaging with the resources (i.e., 1 month after Time 3), then significant changes may have been observed. The idea that changes in hope may not be immediate is supported by findings from a recent systematic review of self-management interventions for people living with severe mental illness [30]. This review found no significant difference in change in hope scores between treatment and control groups at the end of treatment (2 studies, *n* = 389, *p* = 0.07) but a significant difference favouring the intervention group at follow-up (3 studies, *n* = 967, *p* = 0.03).

It is also possible that the hope scores for Time 1 were artificially inflated through the process of recruitment and consent relating to the research project. Previous research has found that two experiences that contribute to hope are: feeling respected, listened to and believed; and contributing or helping others [9]. Our qualitative data suggests that people may have derived hope from finding out about lived experience research and being asked to take part in the research project. People felt that their views and experiences were being valued and could see that by participating in the project they were contributing to something that may help others in the future. It may well be that levels of hope, if measured before the project was explained to participants (a hypothetical possibility only) may have been lower, suggesting that the change between Time 1 and Time 2 that relates to the resources may be underestimated. Given that hope is an overall feeling about life, which is influenced by many factors, the finding that hopefulness increased overall within the short timeframe of our small study suggests a potential benefit of lived experience research that should be further investigated.

While participants’ reports of the impact of the lived experience resources on their lives were very positive, there were a couple of instances where a participant reported a negative impact. This was despite the involvement of peer workers and other people with lived experience in resource development and our efforts to present positive and empowering perspectives. In each case, the negative experience did appear to be within the context of a wider positive experience with the resources. However, given that every individual’s situation and history is unique, it may be impossible to ensure that a resource will never cause distress. Further, short term discomfort may sometimes be ultimately productive. Shifts in perspectives and understandings can often involve tension and conflict as people grapple with new ways of thinking and what these might mean for their previously held stories e.g., [31]. The findings suggest the importance of involving, in dissemination of such resources, peer workers or others who have a relationship with the person and are experienced in dealing with these kinds of issues, and potential distress. For people who are vulnerable, it may be advisable for peer workers to go through the resource with them, rather than presenting it as a stand-alone resource, while for others it may be advisable to check in with people about their reactions. This issue and the role of peer workers is discussed in detail elsewhere [Authors, in preparation].

When searching for research to use for this study, it was more difficult than anticipated to find appropriate studies. This was for two main reasons. First, there are no standard keywords to identify lived experience research and authors do not always declare their lived experience status. Anecdotal evidence indicates that the latter may be a reflection of stigma and potential discrimination in publishing. Second, we found that only a small minority of lived experience research suggested implications that could be used directly in people’s daily lives. Rather, most was aimed at increasing the understanding or changing the behaviour of health professionals and policy makers [32]. This type of research is clearly important. However, the current study highlights the potential usefulness of lived experience research focused on facilitating positive knowledge, attitudes and strategies for services users. It suggests the need for funding bodies and publishers to support lived experience research that will produce findings that can be used directly in people’s daily lives. The current study contributes to knowledge translation by highlighting a strategy that addresses the problem of accessing the evidence base and rendering that evidence base user friendly [33].

This study has several limitations. As with any study relying on volunteers, it is possible that participants were, at the outset, more positively inclined toward lived experience research than those who declined to participate. Peer workers may also have inadvertently differentially approached people they thought would enjoy or benefit from the resources. The sample size was quite small so, for the analysis of change in hope, it is possible that some real differences may not have been identified. A further limitation of the study is that 36 of the 38 participants were living in the community. While peer workers believed that many of the resources would be useful in inpatient settings, logistical and staff issues meant that recruitment was primarily from the community. Future research is needed to confirm the findings of this study with a wider sample, including people in a variety of mental health settings.

It should be acknowledged that this study did not compare resources developed from lived experience research to similar resources developed from other research that was designed to illuminate lived experience perspectives but was not conducted by researchers with their own lived experience. Therefore, while a number of participants expressed positive feelings about the research being done by people with lived experience, it is still unclear to what extent the lived experience authorship was critical to participants’ engagement with the resources.

It is also important to recognise that participants engaged with the resources, not simply as part of their everyday interactions with their peer workers, but in the context of a research project. Participants’ positive experiences with being part of the research project may have affected their overall reactions to the resources. It was impossible to disentangle participants’ experiences of the resources themselves from their experiences of being a participant whose opinions and experiences were being sought for a research study which ultimately aimed to help improve the lives of other people who experience mental health issues. The authors are currently designing a project to investigate the use of the resources in peer workers’ routine practice. By offering resources and training to a large sample of peer workers, then allowing them to use the resources where they feel it is appropriate, we will get a clearer sense of the usefulness of these resources in everyday practice.

## Conclusions

Many benefits have been acknowledged in recent years of mental health research being conducted by or in collaborations including researchers with lived experience, for both the researchers and the research itself e.g., [1, 6]. The current research indicates that lived experience research, when brought to their attention and presented in user-friendly formats, also has the potential to provide direct benefits to people living with mental health issues. By advocating for lived experience research and sharing the findings in accessible ways, researchers, peer workers and others can support people living with mental health issues to develop new knowledge that they can use for their self-empowerment, recovery and wellbeing.

## Supplementary information

**Additional file 1.**

**Additional file 2.**

**Additional file 3.**

## Data Availability

The dataset for the qualitative interviews analysed during the current study are not publicly available as they consist of audio files and transcripts from in-depth interviews which, even with pseudonyms, might potentially allow individual participants to be identified. Deidentified data from the quantitative analysis are available from the corresponding author on reasonable request.

## Abbreviations

LHD: Local Health District
HHI: Herth Hope Index.
