# Supplementary material for: Lived experience research as a resource for recovery: a mixed methods study
Source: BMC Psychiatry. 2020 Sep 21;20:456. doi: 10.1186/s12888-020-02861-0 (PMC7507671; doi:10.1186/s12888-020-02861-0)
Supplement: Supplementary file 1 — Additional file 1. [file 12888_2020_2861_MOESM1_ESM.pdf]

DESCRIPTION OF RESOURCES

Concepts of Recovery Podcast

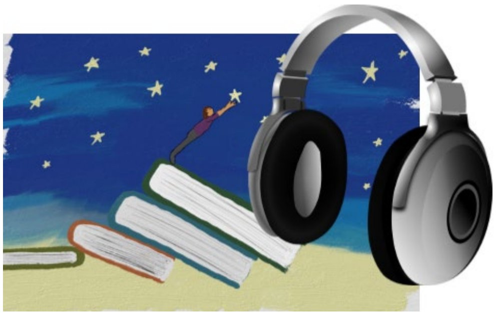

Based on: *Tooth, B. A, Kalyanasundaram, V, Glover, H & Momenzidah (2003). Factors consumers identify and important to recovery from schizophrenia. Australasian Psychiatry 11 (1), 70-77. DOI: 10.1046/j.1440-1665.11.s1.1.x*

Tooth, Kalyanasundaram, Glover and Momenzadah (2003) conducted interviews with 57 people who self-identified as being in recovery to identify what they considered as important in this journey. Thematic analysis was used to identify common themes. These themes were not diagnosis specific but likely to be useful to anyone living with mental health issues. The STELLER research project created a 34-minute podcast of an interview with two of the lead authors, Dr. Barbara Tooth and Ms. Helen Glover. They discussed what inspired them to conduct the study, their experiences of the research, the findings, and their own thoughts about recovery and what it means.

What Helps Recovery

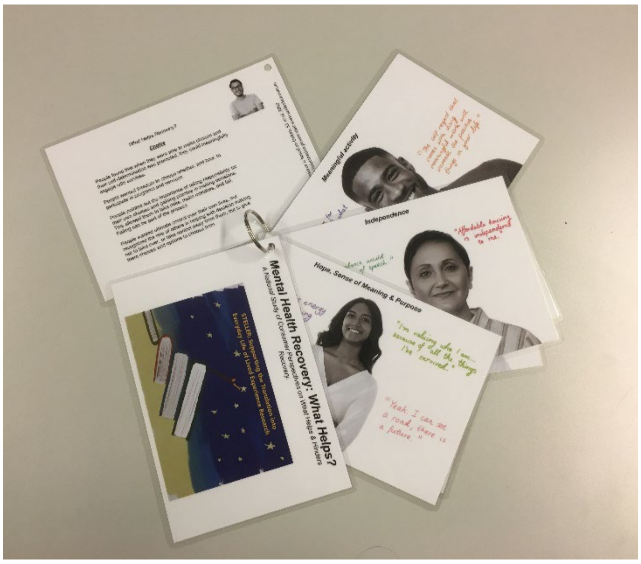

Based on: *Onken, S.J., Dumont, J.M., Ridgway, P., Dornan, D.H., & Ralph, R.O. (2002). Mental health recovery: What helps and what hinders? A national research project for the development of recovery facilitating system performance indicators. Phase one research report: A national study of consumer perspectives on what helps and hinders mental health recovery. National Association of State Mental Health Program Directors (NASMHPD) National Technical Assistance Center (NTAC), Alexandria, VA*

Onken, Dumont, Ridgway, Dornan and Ralph (2002) conducted structured focus groups with 115 people to understand what helped or hindered their recovery. The STELLER research project focussed on the findings for “what helps” to create two variations of a resource entitled “Mental Health Recovery: What Helps?”. One version of the resource was developed to be viewed like an art exhibition with seven A3 sized prints. Each contained a black and white photograph of a person (stock image) along with direct quotes from the research handwritten in different colours. Next to the print was a small placard, as would also be found at an art gallery, containing the title of the theme and a description of what the research said on that theme. For example, one theme was “Hope, Sense of Meaning & Purpose” and a quote included in hand-written style text (in green) was “I’m valuing who I am… because of all the things I’ve survived.” A smaller version of the resource was created for people who did not view the resources at the mental health unit. These were A5 laminated cards put together with a metal ring, so users could flip through the content and open the ring to remove cards of interest to display.

Personal Medicine

Based on: *Deegan, P. (2005). The importance of personal medicine: A qualitative study of resilience in people with psychiatric disabilities. Scandinavian Journal of Public Health, 33(66_suppl), 29–35. DOI: 10.1080/14034950510033345*

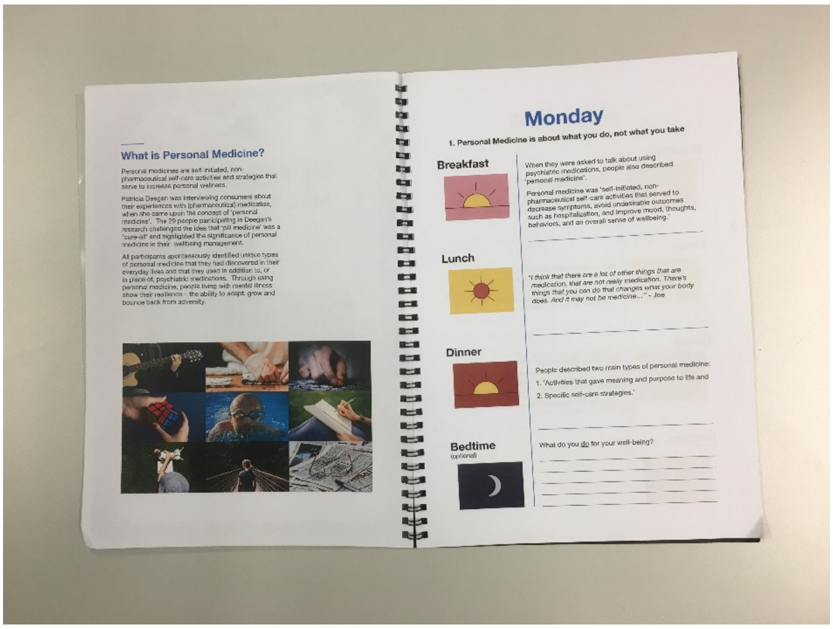

Deegan (2005) used a qualitative method and participatory action design following interviews with 29 people about their experiences with pharmaceutical medication. Findings revealed the concept of 'personal medicine', described as "self-initiated, non-pharmaceutical self-care activities and strategies" that serve to increase personal wellness. The STELLER research project used findings from this study to create a resource entitled the "Personal Medicine Pack", modelled on a webster pack (or pill box). The resource was an A4 booklet divided into the seven days of the week. For each day, the booklet included information and direct quotes from the study as well as prompts to encourage people to think about their own personal medicine. For example, the Monday page, entitled "Personal Medicine is about what you do, not what you take", introduced the concept, explained what personal medicine is and the two types of personal medicine, provided a quote from a participant, and asked the reader to consider what things they did for their well-being. At the end of the book were three 'personal medicine self-prescription' templates, adapted from the Living Edge KIT (part of the Living Edge program, Queensland).

<u>Hope Box</u>

Based on: *Yeung, W., Hancock, N., Honey, A., Wells, K., & Scanlan, J. (2020). Igniting and maintaining hope: The voices of people living with mental illness. Community Mental Health Journal, 56(6), 1044–1052. doi: 10.1007/s10597-020-00557-z*

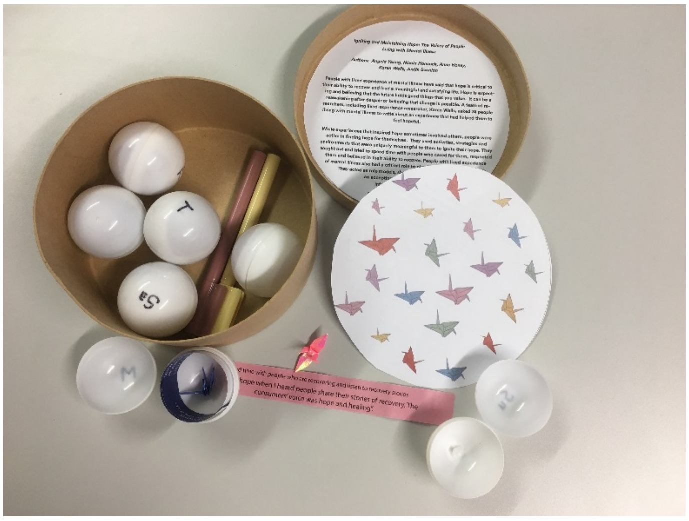

Yeung, Hancock, Wells, Honey and Scanlan (2020) asked 74 people living with mental health issues to write about an experience that had helped them to feel hopeful. They analysed responses using interpretive content analysis. The STELLER research project translated some of these findings into a unique resource we called the hope box. The hope box is a plain, circular cardboard box which contains (1) a brief overview of the study; (2) an insert summarising the classic story of hope and perseverance “Sadako and the Thousand Paper Cranes”; (3) several coloured textas to encourage participants to make the box their own by decorating or writing on it and, most importantly; (4) seven small white hollow balls labelled with a letter, indicating a day of the week. While participants were told to use the resource however they liked, opening one ball each day was a suggestion. Each ball contained a tiny hand-made colourful paper crane and a slip of paper with a key concept and quotes from the study. For example, one insert read: Acknowledge your strengths and your positive progress! “See how far I’ve come since first being diagnosed”, “I’ve many other strengths including being an insightful, gracious, kind, compassionate, brave and determined person.”

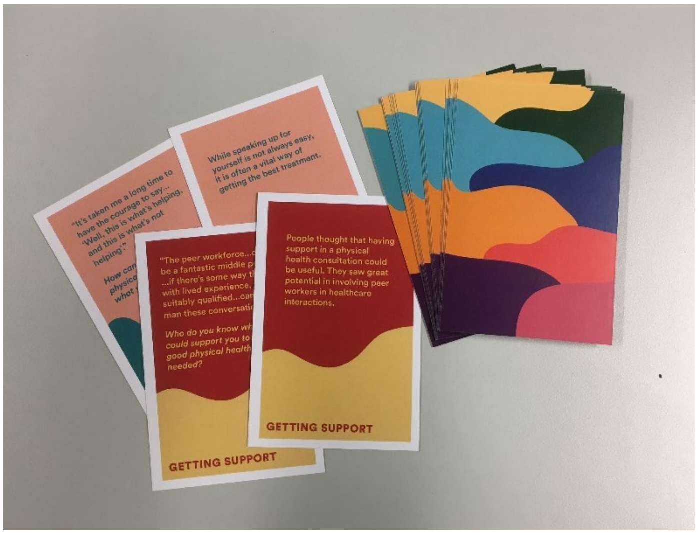

Physical Healthcare Cards

Based on: *Ewart, S., Bocking, J., Happell, B., Platania-Phung, C., & Stanton, R. (2016). Mental health consumer experiences and strategies when seeking physical health care: A focus group study. Global Qualitative Nursing Research, 3, 1-10. 2333393616631679. DOI: 10.1177/2333393616631679*

Ewart, Bocking, Happell, Platania-Phung and Stanton (2016) conducted focus groups with 31 people living with mental health issues to find out about their experiences using health services for their physical health needs, as this group often do not get the same level of physical health care that other people do. Findings included information about what people did to get better care. The STELLER research project created a set of 'physical health care cards' based on these findings. The resource was a pack of 26 cards, with a colourful design on the back, similar in size to a normal deck of cards. The graphically designed front of the cards were colour matched in pairs to allow them to be used to play a memory game. The first four cards included an introduction, overview of the study and suggested use of the resource. The remainder of the cards were separated into eleven themes (two cards per theme), with unique colours representing each theme. One card in each pair provided a description of the theme and the other included direct quotes. Reflective questions were also sometimes included. For example, the "taking time" pair included: "People said that it was important to allow enough time in an appointment to fully deal with physical concerns"; and "One person said that if he was feeling hurried or rushed, he would say: 'Well hang on a minute. I need help with this. Can you refer me to these other services?'. What else might you do or say to make sure you get the time you need with a health professional?".

Meaningful Activities Magazine

Based on: *Biringer, E., Davidson, L., Sundfør, B., Lier, H.Ø., & Borg, M. (2016) Coping with mental health issues: Subjective experiences of self-help and helpful contextual factors at the start of mental health treatment, Journal of Mental Health, 25:1, 23-27, doi: 10.3109/09638237.2015.1078883*

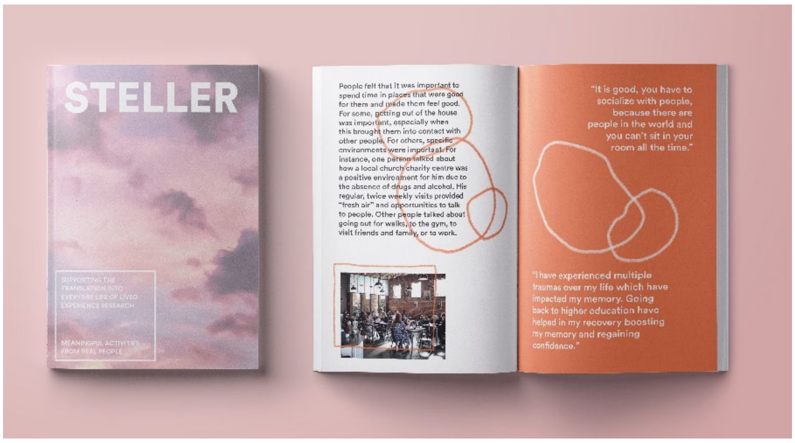

Biringer, Davidson, Sundør, Lier and Borg (2016) conducted in-depth interviews with ten mental health service users in the early stages of treatment (0-7 years). They used a collaborative-reflexive framework to explore what they experienced as helpful and what they had done to help themselves. Analysis revealed that one of the most important things they did was engage in activities each day that were meaningful to them. The STELLER research project translated key information from this study into the 'meaningful activities magazine'. The magazine was A5 in size and was 29 pages. It included an 'editor's note' with an overview of the study, followed by concepts and direct quotes from the study about different types of meaningful activities and how people used them. Additional quotes were added from other lived-experience sources. These were presented in attractive and varied text with colourful pictures, images and backgrounds created by a graphic designer. For example, one section was about music, "making a conscious choice to listen to happy music as that is a good way of changing how you feel". The magazine also included a "brain teasers" section with a word list and Sudoku game, a section for mindful colouring and two blank pages at the end to list or sketch activities that are most meaningful to the reader.
