# Supplementary material for: Lived experience research as a resource for recovery: a mixed methods study
Source: BMC Psychiatry. 2020 Sep 21;20:456. doi: 10.1186/s12888-020-02861-0 (PMC7507671; doi:10.1186/s12888-020-02861-0)
Supplement: Supplementary file 2 — Additional file 2. [file 12888_2020_2861_MOESM2_ESM.pdf]

Your experience with the lived-experience research resources

Please complete the survey below, to tell us how you experienced the lived-experience research resources.

Your answers to this survey are anonymous.

Which lived-experience research resources did you see throughout the study?

- ☐ Concepts of recovery (the podcast)
- ☐ What helps recovery (the portraits)
- ☐ Hope (the hope box)
- ☐ Personal medicine (the webster pack booklet)
- ☐ Looking after physical health (the card pack)
- ☐ Meaningful activity (the magazine)

Think about the 'concepts of recovery' resource (the podcast).

How much time did you spend listening to the 'concepts of recovery' resource?

- ◯ No time
- ◯ Less than 5 minutes
- ◯ 5-10 minutes
- ◯ 10-20 minutes
- ◯ More than 20 minutes

How interesting did you find the resource?

- ◯ Not interesting
- ◯ A bit interesting
- ◯ Very interesting
- ◯ Extremely interesting

How easy was it to understand?

- ◯ Very difficult
- ◯ A bit difficult
- ◯ Fairly easy
- ◯ Very easy

	Not helpful	A bit helpful	Very helpful	Extremely helpful
How helpful did you find that resource?	◯	◯	◯	◯

Has the 'concepts of recovery' resource made a difference in your life?

	A big improvement	A small improvement	No difference	Worsened	Not sure
Your feelings about yourself	◯	◯	◯	◯	◯
Your perspective on your own experiences	◯	◯	◯	◯	◯
Your beliefs about your future or recovery	◯	◯	◯	◯	◯
Your ability to care for yourself and your mental health	◯	◯	◯	◯	◯

Your ability to deal with the mental health system	◯	◯	◯	◯	◯
Your ability to interact with mental health professionals	◯	◯	◯	◯	◯
Your understanding of other people and their experiences	◯	◯	◯	◯	◯
Your understandings about mental illness	◯	◯	◯	◯	◯
Another sort of difference	◯	◯	◯	◯	◯

Please describe this difference.

____

Would you recommend the resource to others?
◯ Yes
◯ No

Think about the 'what helps recovery' resource (the portraits).

How much time did you spend looking at the 'what helps recovery' resource?
◯ No time
◯ Less than 5 minutes
◯ 5-10 minutes
◯ 10-20 minutes
◯ More than 20 minutes

How interesting did you find the resource?
◯ Not interesting
◯ A bit interesting
◯ Very interesting
◯ Extremely interesting

How easy was it to understand?
◯ Very difficult
◯ A bit difficult
◯ Fairly easy
◯ Very easy

	Not helpful	A bit helpful	Very helpful	Extremely helpful
How helpful did you find that resource?	◯	◯	◯	◯

Has the 'what helps recovery' resource made a difference in your life?

	A big improvement	A small improvement	No difference	Worsened	Not sure
Your feelings about yourself	◯	◯	◯	◯	◯
Your perspective on your own experiences	◯	◯	◯	◯	◯
Your beliefs about your future or recovery	◯	◯	◯	◯	◯
Your ability to care for yourself and your mental health	◯	◯	◯	◯	◯
Your ability to deal with the mental health system	◯	◯	◯	◯	◯
Your ability to interact with mental health professionals	◯	◯	◯	◯	◯
Your understanding of other people and their experiences	◯	◯	◯	◯	◯
Your understandings about mental illness	◯	◯	◯	◯	◯
Another sort of difference	◯	◯	◯	◯	◯

Please describe this difference. ________________________________

Would you recommend the resource to others?
- ◯ Yes
- ◯ No

Think about the 'hope' resource (the hope box).

How much time did you spend with the 'hope' resource?
- ◯ No time
- ◯ Less than 5 minutes
- ◯ 5-10 minutes
- ◯ 10-20 minutes
- ◯ More than 20 minutes

How interesting did you find the resource?
- ◯ Not interesting
- ◯ A bit interesting
- ◯ Very interesting
- ◯ Extremely interesting

How easy was it to understand?
- ◯ Very difficult
- ◯ A bit difficult
- ◯ Fairly easy
- ◯ Very easy

	Not helpful	A bit helpful	Very helpful	Extremely helpful
How helpful did you find that resource?	◯	◯	◯	◯

Has the 'hope' resource made a difference in your life?

	A big improvement	A small improvement	No difference	Worsened	Not sure
Your feelings about yourself	◯	◯	◯	◯	◯
Your perspective on your own experiences	◯	◯	◯	◯	◯
Your beliefs about your future or recovery	◯	◯	◯	◯	◯
Your ability to care for yourself and your mental health	◯	◯	◯	◯	◯
Your ability to deal with the mental health system	◯	◯	◯	◯	◯
Your ability to interact with mental health professionals	◯	◯	◯	◯	◯
Your understanding of other people and their experiences	◯	◯	◯	◯	◯
Your understandings about mental illness	◯	◯	◯	◯	◯
Another sort of difference	◯	◯	◯	◯	◯

Please describe this difference.

____

Would you recommend the resource to others?

◯ Yes
◯ No

Think about the 'personal medicine' resource (the webster pack booklet).

How much time did you spend with the 'personal medicine' resource?

◯ No time
◯ Less than 5 minutes
◯ 5-10 minutes
◯ 10-20 minutes
◯ More than 20 minutes

How interesting did you find the resource?

◯ Not interesting
◯ A bit interesting
◯ Very interesting
◯ Extremely interesting

How easy was it to understand?

◯ Very difficult
◯ A bit difficult
◯ Fairly easy
◯ Very easy

	Not helpful	A bit helpful	Very helpful	Extremely helpful
How helpful did you find that resource?	◯	◯	◯	◯

Has the 'personal medicine' resource made a difference in your life?

	A big improvement	A small improvement	No difference	Worsened	Not sure
Your feelings about yourself	◯	◯	◯	◯	◯
Your perspective on your own experiences	◯	◯	◯	◯	◯
Your beliefs about your future or recovery	◯	◯	◯	◯	◯
Your ability to care for yourself and your mental health	◯	◯	◯	◯	◯
Your ability to deal with the mental health system	◯	◯	◯	◯	◯
Your ability to interact with mental health professionals	◯	◯	◯	◯	◯
Your understanding of other people and their experiences	◯	◯	◯	◯	◯
Your understandings about mental illness	◯	◯	◯	◯	◯
Another sort of difference	◯	◯	◯	◯	◯

Please describe this difference.

____

Would you recommend the resource to others?

◯ Yes
◯ No

Think about the 'looking after physical health' resource (the card pack).

How much time did you spend with the 'looking after physical health' resource?

◯ No time
◯ Less than 5 minutes
◯ 5-10 minutes
◯ 10-20 minutes
◯ More than 20 minutes

How interesting did you find the resource?

◯ Not interesting
◯ A bit interesting
◯ Very interesting
◯ Extremely interesting

How easy was it to understand?

◯ Very difficult
◯ A bit difficult
◯ Fairly easy
◯ Very easy

	Not helpful	A bit helpful	Very helpful	Extremely helpful
How helpful did you find that resource?	◯	◯	◯	◯

Has the 'looking after physical health' resource made a difference in your life?

	A big improvement	A small improvement	No difference	Worsened	Not sure
Your feelings about yourself	◯	◯	◯	◯	◯
Your perspective on your own experiences	◯	◯	◯	◯	◯
Your beliefs about your future or recovery	◯	◯	◯	◯	◯
Your ability to care for yourself and your mental health	◯	◯	◯	◯	◯
Your ability to deal with the mental health system	◯	◯	◯	◯	◯
Your ability to interact with mental health professionals	◯	◯	◯	◯	◯
Your understanding of other people and their experiences	◯	◯	◯	◯	◯
Your understandings about mental illness	◯	◯	◯	◯	◯
Another sort of difference	◯	◯	◯	◯	◯

Please describe this difference.

____

Would you recommend the resource to others?

◯ Yes
◯ No

Think about the 'meaningful activity' resource (the magazine).

How much time did you spend with the 'meaningful activity' resource?

◯ No time
◯ Less than 5 minutes
◯ 5-10 minutes
◯ 10-20 minutes
◯ More than 20 minutes

How interesting did you find the resource?

◯ Not interesting
◯ A bit interesting
◯ Very interesting
◯ Extremely interesting

How easy was it to understand?

◯ Very difficult
◯ A bit difficult
◯ Fairly easy
◯ Very easy

	Not helpful	A bit helpful	Very helpful	Extremely helpful
How helpful did you find that resource?	◯	◯	◯	◯

Has the 'meaningful activity' resource made a difference in your life?

	A big improvement	A small improvement	No difference	Worsened	Not sure
Your feelings about yourself	◯	◯	◯	◯	◯
Your perspective on your own experiences	◯	◯	◯	◯	◯
Your beliefs about your future or recovery	◯	◯	◯	◯	◯
Your ability to care for yourself and your mental health	◯	◯	◯	◯	◯
Your ability to deal with the mental health system	◯	◯	◯	◯	◯
Your ability to interact with mental health professionals	◯	◯	◯	◯	◯
Your understanding of other people and their experiences	◯	◯	◯	◯	◯
Your understandings about mental illness	◯	◯	◯	◯	◯
Another sort of difference	◯	◯	◯	◯	◯

Please describe this difference. __________________________________

Would you recommend the resource to others?
- ◯ Yes
- ◯ No

And a couple of final questions about your experience as a whole.

Are you interested in accessing more lived-experience research in the future?
- ◯ No
- ◯ Probably not
- ◯ Maybe
- ◯ Definitely

Have you shared your knowledge from lived-experience research with anyone else?
- ◯ Yes
- ◯ No
- ◯ Not sure

Overall, how would you describe your experience of participating in the study?
- ◯ Negative
- ◯ Neutral
- ◯ Quite positive
- ◯ Very positive

Please tell us anything else that will help us to understand your experience or improve lived-experience resources in the future. __________________________________
