# Supplementary material for: Lived experience research as a resource for recovery: a mixed methods study
Source: BMC Psychiatry. 2020 Sep 21;20:456. doi: 10.1186/s12888-020-02861-0 (PMC7507671; doi:10.1186/s12888-020-02861-0)
Supplement: Supplementary file 3 — Additional file 3. [file 12888_2020_2861_MOESM3_ESM.pdf]

Making lived experience research accessible in recovery

Qualitative interview guideline questions

Can you tell me a little bit about your overall experience as a participant in this research?

- Why did you choose to be involved/what attracted you to the study?
- Do you think you got any benefits from being in the study?
- Was there anything that you did not like about being in the study?
- What could the research team have done differently to make the research experience better? Before/during/after the project

What are your thoughts about the lived experience research resources? (Provide a summary of the 4 different resources.)

Overall experience

- Which resource did you like best? And why?
- Which resource did you like least? And why?
- How long did you spend with each resource? Was this enough time/too much/too little?

Content

- What information from the resources do you remember most? Why?
- Did you find some information more useful than other information? Why?

Format

- Was any particular format more effective at sharing lived experience? Was any one more powerful? Why?
- Are there other types of resources that you think should be considered to share lived experience?

Thinking back on your involvement in the study, can you talk a little bit about the usefulness of lived experience to you?

- Did anything change for you as a result of engaging with these resources?

  [e.g., learn or think differently, feel differently, do something different, share with others, help/hinder day-to-day coping? enhance/inspire a sense of hope]

NB: Because this is a semi-structured interview, these questions are guidelines only. The interviewer may use different wording, ask the questions in a different order, and follow up on what is important to the interviewee. The guide may also be modified to address issues that become salient throughout the research process, or in prior interviews.
